# Increased mortality in patients with non-functioning pituitary tumors: a study in a tertiary center

**DOI:** 10.3389/fendo.2025.1653376

**Published:** 2025-08-04

**Authors:** Guadalupe Vargas-Ortega, Edgar-Manuel Ramírez-García, Lourdes Balcázar-Hernández, Mario Enrique Rendón-Macias, Carlos Alfonso Romero-Gameros, Baldomero González-Virla, Blas López-Félix, Erick Zepeda, Eric Misael Estrada-Estrada

**Affiliations:** ^1^ Endocrinology Department, Hospital de Especialidades, Centro Médico Nacional Siglo XXI, Instituto Mexicano del Seguro Social, Mexico City, Mexico; ^2^ Department of Public Health, Faculty of Health Sciences, Universidad Panamericana, Mexico City, Mexico; ^3^ Otorhinolaryngology Department, Hospital Ángeles Ciudad Juárez, Ciudad Juárez, Mexico; ^4^ Neurosurgeon Department, Hospital de Especialidades, Centro Médico Nacional Siglo XXI, Instituto Mexicano del Seguro Social, Mexico City, Mexico

**Keywords:** non-functioning pituitary adenomas, NFPA, mortality, pituitary surgery, arterial hypertension, diabetes

## Abstract

**Background information:**

Most of the studies on mortality, survival analysis and predictor variables in patients with non-functioning pituitary adenomas (NFPA) are heterogeneous and limited; and in some cases, they offer contradictory results.

**Patients and methods:**

Our objective was to analyze the survival of 749 patients (46.3% were women) with NFPA, with a mean follow-up of 60.2 months (32.9-120.6) at a tertiary center, from March 2007 to August 2023. Descriptive and inferential statistics were used. Mortality risk was determined in comparison with the Mexican population by calculating the standardized mortality ratio (SMR) with 99% confidence intervals. Survival analysis and Cox proportional hazards (HR) analysis were performed to identify possible predictors.

**Results:**

The overall SMR was of 8.86 (99% Confidence Interval [CI] 5.92-13.25, p<0.001), for <60 years-old patients, it was 13.77 (99% CI 7.23-26.23, p<0.001). In men, it was 7.08 (99% CI 3.92-12.80, p<0.001), and in women it was 11. 11 (99% CI 6.41-19.24, p<0.001). Regarding patients with arterial hypertension, we obtained 6.59 (99% CI 3.75-11.56, p<0.001), and with type 2 diabetes, it was 4.63 (99% CI 1.96-10.93, p<0.001). In the Cox analysis for mortality, an adjusted HR of 2.19 (95% CI 1.13-4.26, p=0.02) was observed for arterial hypertension and 1.32 (95% CI 1.00-1.75, p=0.04) for the total number of surgeries. The rest of the variables had no statistically significant association with mortality.

**Conclusion:**

Our study shows a higher mortality in patients diagnosed with NFPA compared to the general population. The higher probability of death was found in the presence of predictors such as arterial hypertension and the total number of surgeries.

## 1 Introduction

Pituitary tumors account for 15% of intracranial tumors (1). Non-functioning pituitary tumors (NFPA) are benign neoplasms that originate from the epithelial cells of the anterior pituitary and are not associated with hormone-hypersecretion syndromes. They account for 15-37% of all pituitary tumors, with a prevalence ranging from 7-11/100,000 inhabitants. Most come with symptoms such as headache, campimetric deficits or ophthalmoplegia caused by the mechanical compression of the tumor mass on the structures surrounding the pituitary gland (2–4). In 67-90% NFPA patients, these are macroadenomas, with an average age ranging from 51.5 to 65.5 years (5, 6). Transsphenoidal surgery is the treatment of choice. However, in some patients, transcranial approaches and postoperative radiotherapy are required if a tumor remnant persists and/or recurs (1). Medical treatment with dopaminergic agonists (DA) is an option in cases where macroadenoma surgery is contraindicated or the tumor remnant is inoperable (7, 8).

The presence of significant morbidity associated with the biological behavior of the tumor (recurrence or persistence rate of 49%) (5) is not uncommon in patients with NFPAs. In an attempt to demassify most of the tumor, its surgical treatment (and the number of surgeries performed) causes compressive data which leads to visual deficits, ophthalmoplegia, headache and endocranial hypertension (1, 5).

Additionally, this increased morbidity in patients with NFPA has also been related to infections due to the surgical procedure, prolonged hospital stays, treatment with radiotherapy (RT) in its different modalities, panhypopituitarism [hormone deficiencies: growth hormone (GH) in 78-88%, follicle stimulating (FSH) and luteinizing (LH) hormones in 46-90%, thyroid stimulating hormone (TSH) in 12-57%, adrenocorticotropic hormone (ACTH) in 13-60% and diabetes insipidus (DI) in 15%; more prevalent in patients treated with RT], increased coronary ischemic disease, hypertension, diabetes and dyslipidemia (9–11). However, most of the published studies on risk factors for morbidity and mortality in these patients are very heterogeneous in their methodologies, the tumors analyzed (they include both pituitary and hypothalamic tumors), the number of individuals included and the selection criteria in the survival analyses (12). Therefore, studies on long-term mortality of patients with NFPA are very limited and with contradictory results [eg, a reported increase in mortality among women (13, 14) versus no observed increase (12, 15), and higher mortality linked to radiotherapy in some series (11, 13, 15) but not in others (14, 16)]. Moreover, some predictors of mortality have been inconclusive and not available in all studies (eg, sex, age at diagnosis, RT), and some important factors such as the impact of hypopituitarism and its treatment (particularly with hormone replacement) have not been clearly defined regarding their impact on survival (12).

The aim of our study was to analyze survival in patients with NFPA in our clinic between March 2007 to August 2023 and to define its possible predictor variables in a well-characterized cohort followed-up in a tertiary hospital.

## 2 Study design

A retrospective historical cohort of NFPA-patients survival at a tertiary care center, with exploratory analysis to identify risk factors associated with mortality.

### 2.1 Patients and methods

The NFPA clinic has been a Patient Care Center since 2007. A retrospective evaluation of the clinical records of patients with NFPA followed between March 2007 and August 2023 was performed. The study was approved by the Local Ethics and Research Committee with Institutional Registry identifier R-2019-3601-032 and adhered to the ethical guidelines of the Declaration of Helsinsky and the Mexican General Health Law on research for health studies. All patients were diagnosed and treated according to a protocol that included: complete clinical history, measurement of anterior pituitary hormones, computerized examination of visual fields and magnetic resonance imaging (MRI) of the sellar region.

### 2.2 Sampling

The sample was a nonprobabilistic sample of consecutive cases available in the review period.

### 2.3 Hormonal measurements

Hormonal measurements of cortisol, TSH, free thyroxine (FT4), testosterone, estradiol, LH, FSH and prolactin were determined by electrochemiluminescence immunoassay (ECLIA) using Elecsys^®^ kits (Roche Diagnostics GmbH, Mannheim, Germany) compatible with Cobas^®^ E series analyzers. The measurement intervals and precision of the methods were evaluated according to CLSI protocol EP05-A3, as reported by the manufacturer. Data on measurement intervals, intra-assay precision, inter-assay precision and mean concentrations for each hormone are shown in **Supplementary Table 1**.

Meanwhile, hormone measurements of GH and insulin like growth factor 1 (IGF1) were determined by ECLIA using Liaison^®^ kits (DiaSorin S.p.A., Saluggia, Italy) compatible with LIAISON^®^ Analyzers, with a detection limit of up to 820 ng/mL and 1500 ng/mL, respectively. Method precision was evaluated according to CLSI protocol C28-A3, with an intra-assay precision of 1.6% (mean concentration of 3.56 ng/mL) and 4.59% (mean concentration of 189.3 ng/mL), as well as an inter-assay precision of 3.9% (mean concentration of 3.29 ng/mL) and 4.3% (mean concentration of 202.6 ng/mL), respectively.

Central hypocortisolism was defined as a morning serum cortisol <5μ g/dL. The diagnosis of central hypothyroidism was based on the finding of a FT4 <0.6 ng/dL in the presence of low or inappropriately normal TSH. Hypogonadotropic hypogonadism was diagnosed when estradiol or testosterone levels were <20 pg/mL and 300 ng/dL, respectively, in the presence of normal or low LH and FSH levels. Prolactin (PRL) measurements were performed in previously diluted sera (1:100) to rule out underestimation by hook effect. Panhypopituitarism was defined as the presence of three or more pituitary hormone deficiencies (1, 17, 18).

### 2.4 Radiological evaluations

All patients underwent 3T MRI preoperatively and postoperatively, usually 6 months after surgery. Tumor volume was calculated with the Di Chiro and Nelson formula. Postoperative remission was considered for those operated for the first time and without subsequent intervention due to the absence of tumor remnant (19).

### 2.5 Multimodal treatment of NFPAs

The main treatment was surgical, performed by three pituitary neurosurgeons (BLF, EZ and EE) in a microscopic or endoscopic transsphenoidal surgical approach; and in some cases, translabial and sublabial according to the location and size of the adenoma. The transcranial approach was reserved for patients with giant and invasive tumors (>4cm). Immunohistochemical analysis was available in <20% of patients and included immunostaining for pituitary transcription factor 1 (POU1F1), steroidogenic factor 1 (NR5A1), estrogen receptor alpha 1 (ESR1), LH, FSH, ACTH, GH, PRL and TSH.

#### 2.5.1 Medical treatment

Patients with postoperative remnant, active growth and contraindication for new surgery or who refused surgical treatment were treated with cabergoline at an initial dose of 1.5 mg per week; followed by an increase up to a ceiling dose of 3 mg per week, or until tumor containment and/or shrinkage was induced.

#### 2.5.2 Treatment with radiotherapy

Three-dimensional conformal external beam radiation therapy was delivered by a linear accelerator at a total dose of 52 Gy (range 50-57), in daily fractions of 2-2.5 Gy, 5 days a week for 5 weeks. The radiation dose from the optical device was kept below 50.4 Gy using a multileaf collimator. In the case of tumors< 3 cm in diameter and located at a minimum distance from the optic chiasm> 3mm, it was decided to use the radiosurgery technique. Radiosurgery was administered using a CyberKnife M6 platform, with the Multi-Plan system, to develop the planning treatments (Accuray Incorporated Sunnyvale, Sunnyvale, CA, USA) for all treatments in Mexico City, at the Oncology Hospital of the *Centro Médico Nacional*. After a patient was accepted to be treated, the MRI and computed tomography (CT) were obtained for the planning. The median radiation dose was of 23.5 Gy (range 22-25 Gy), delivered in a single day, or a maximum of five days, in 5 Gy to 22 Gy per fraction. The individual Radiosurgery radiation protocol was decided by the radiation oncologist and neurosurgeon, based on the availability of appointments and the specific circumstances of each patient. For instance, if patients lived out of town, the Institution provided accommodation for the duration of their treatments. Different organs at risk were carefully protected, and all passed the Normal Tissue Constraints, according to the R.D. Timmerman charts. Medical treatments with DA were suspended at least one month before and during radiotherapy.

### 2.6 Patient follow-up

Patients were taken into consideration as of the date of their admission to our center for diagnostic confirmation and until death from any cause (reported in the clinical record), or completion of the study.

### 2.7 Statistical analysis

Descriptive and inferential statistics were used for data analysis. Qualitative variables are summarized as simple and relative frequencies in percentages. Quantitative variables are summarized as medians and interquartile range Q1-Q3.

To determine the mortality risk in comparison with the Mexican population without NFPA, the standardized mortality ratio (SMR) with its 99% confidence intervals (CI 99%) was estimated. For its calculation, we obtained the general mortality rates by sex, age (>18 years) and comorbidities (systemic arterial hypertension and type 2 diabetes) published by the *Instituto Nacional de Estadística y Geografía* (INEGI) of the Government of Mexico, from the 2023 report (20–23). With these rates, we estimated the expected deaths in our patient population by the indirect method. The SMRs were estimated by dividing the observed cases by the expected cases according to: general population in the >18 years age group, in the >60 years age group, by sex, and by history of arterial hypertension or type 2 diabetes. Confidence intervals were calculated assuming a Poisson distribution.

The comparison of baseline characteristics between patients who survived until the study cutoff and those who died was performed by using the Chi-square test for qualitative variables and the Mann Whitney U test for quantitative variables.

Survival analysis was performed in 749 patients. The calculation of the magnitude of the association between time to death with respect to the predictor variables considered was estimated through a crude Cox multivariate proportional hazards analysis; and it was adjusted considering only variables with biological plausibility and statistical significance. A value of p<0.05 was considered to establish statistical significance, in addition to the respective CIs. The statistical package STATA SE (version 17.0; Stata Corp) was used to perform the data analysis.

## 3 Results

### 3.1 Descriptive analysis

During the study period, 749 patients were analyzed (*see* **Table 1**) after an average median follow-up of 60.2 months (min-max: 32.9-120.6). The median age was 60 years (min-max: 49-70) and 46.3% were female. The 62.2% had some family history where type 2 diabetes was the most common (22.4%), followed by arterial hypertension (11.3%). The main comorbidities were: hypertension, dyslipidemia and type 2 diabetes (**Table 1**).

**Table 1:** Baseline characteristics of the 749 patients with non-functioning pituitary adenomas followed-up between the years of 2007-2023.

Variable	n = 749
Female, No. (%)	347 (46.3)
Age, median [IQR] years	60 [49-70]
Age <60, No. (%)	360 (48.1)
Any family history, No. (%)	466 (62.2)
Lag-time at diagnosis, median [IQR] months	9.43 [2.5-21.37]
Comorbidities	
Systemic arterial hypertension, No. (%)	210 (28.0)
Dyslipidemia, No. (%)	205 (27.4)
Diabetes type 2, No. (%)	113 (15.1)
Clinical data	
Campimetric deficit, No. (%)	661 (88.2)
Panhypopituitarism at diagnosis, No. (%)	150 (20)
Giant adenoma, No. (%)	138 (18.4)
Headache, No. (%)	113 (15.1)
Stroke, No. (%)	49 (6.5)
Ophthalmoplegia, No. (%)	28 (3.7)
Endocranial hypertension, No. (%)	23 (3.1)
Incidentaloma, No. (%)	62 (8.3)
Tumor characteristics	
Cephalocaudal diameter, median [IQR] cm	3 [2.5-3.5]
Transverse diameter, median [IQR] cm	2.6 [2.1-3]
Anteroposterior diameter, median [IQR] cm	2.5 [2.1-3]
Tumor volume, median [IQR] cm^3^	10.21 [6.47-14.14]
Invasion to neighboring structures, No. (%)	505 (67.4)
Treatments	
Surgical, No. (%)	525 (70.1)
Radiotherapy, No. (%)	158 (21.1)

With respect to NFPA, the time from initial patient-referred symptom to diagnosis (lag-time) had a median of 9.43 months (min-max; 2.5-21.37). Most of the patients were diagnosed with clinical symptoms where 88.2% presented campimetric deficit (**Table 1**). It should be noted that 18.4% had a giant adenoma. Incidentaloma was observed in 8.3% and 67.4% of the patients had a tumor that invaded neighboring structures (Hardy Knosp IIIB-IV).

Regarding hormonal deficits at diagnosis, 59.1% had hypothyroidism, 30.4% had hypocortisolism and 34.6% had hypogonadism. Panhypopituarism was present in 20% of the patients, while 33.1% did not present hormonal compromise. On the other hand, baseline PRL was 21 ng/dl (10.91-44).

At diagnosis, the median cephalo-caudal tumor diameters were 3 cm (2.5-3.5), transverse 2.6 cm (2.1-3) and anteroposterior 2.5 cm (2.1-3), with a tumor volume of 10.21 cm3 (6.47-14.14).

Regarding surgical treatment, 29.9% (n=224) of the patients did not undergo surgery (due to personal reasons and medical contraindication), 46.7% (n=350) did only once, 9.75% (n=73) twice, 12.15% (n=91) three times and 1.47% (n=11) up to four surgeries. Postoperatively, a cerebrospinal fluid fistula occurred in 6.28%, while vasopressin insufficiency occurred in 17.09% of patients.

Postoperative remission was found in 46.98% of patients. The median final tumor volume after multimodality treatment was 1.443 cm3 (range 0.323 – 1480.4). Recurrence after the first surgery was observed in 32% (n=194) of the patients, of which only 37% (n=73) returned for a second surgery.

Treatment with radiotherapy was offered to 21.1% (n=158) of patients, 82.28% with conventional radiotherapy (fractionated stereotactic) and 17.72% with radiosurgery. For conventional radiotherapy the median number of sessions was 26.5 [25-28].

### 3.2 Mortality analysis

**Figure 1:**
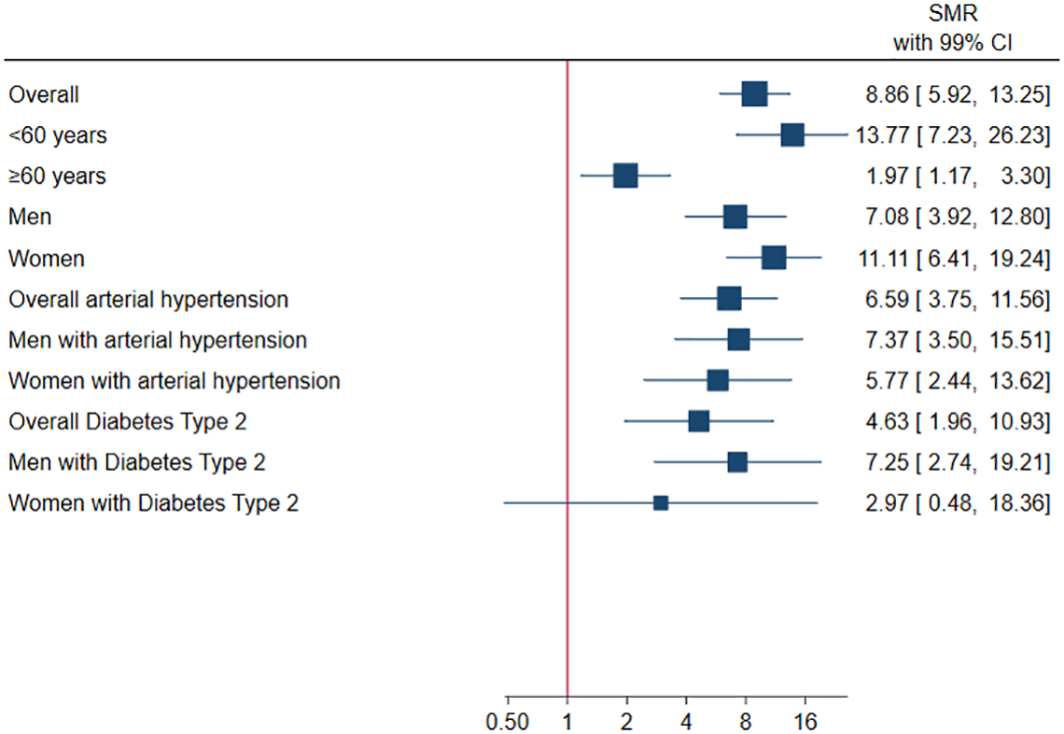
Mortality in non-functioning pituitary adenomas.

A mortality analysis was performed on the 749 patients, of whom 41 died during the study period. When compared to an expected number of deaths of 4.62 in general population of this age group (>18 years), it resulted in an estimated SMR of 8.86 (99% CI 5.92-13.25, p<0.001) (See **Table 2** and **Figure 1**). For patients aged <60 years, the SMR increased to 13.77 (99% CI 7.23-26.23, p<0.001) (*See* **Figure 1**). Comparatively, mortality was higher in women than in men (SMR 11.11, 99% CI 6.41-19.24, p<0.001 vs SMR 7.08, 99% CI 3.92-12.80, p<0.001). In relation to the comorbidities, higher mortality was found in patients with arterial hypertension (SMR 6.59. 99% CI 3.75-11.56, p<0.001), with diabetes type 2 (SMR 4.63, 99% CI 1.96-10.93, p<0.001) and especially in males with both comorbidities (SMR 7.37, 99% CI 3.50-15.51, p<0.0011 vs SMR 7.25, 99% CI 2.74-19.21, p<0.001 respectively).

**Table 2:** Standardized mortality ratio in patients with non-functioning pituitary adenomas.

Mortality	Expected deaths	Deaths observed	SMR (99% CI)	P
General	4.62	41	8.86 (5.92-13.25)	<0.001
Age <60 years old	1.16	16	13.77 (7.23-26.23)	<0.001
Age ≥60 years	12.67	25	1.97 (1.17-3.30)	<0.001
Men	2.68	19	7.08 (3.92-12.80)	<0.001
Women	1.97	22	11.11 (6.41-19.24)	<0.001
Arterial hypertension	3.18	21	6.59 (3.75-11.56)	<0.001
-Men	1.62	12	7.37 (3.50-15.51)	<0.001
-Women	1.55	9	5.77 (2.44-13.62)	<0.001
Type 2 diabetes	1.94	9	4.63 (1.96-10.93)	<0.001
-Men	0.96	7	7.25 (2.74-19.21)	<0.001
-Women	0.67	2	2.97 (0.48-18.36)	0.12

### 3.3 Baseline bivariate analysis

The bivariate analysis was carried out in 749 patients (41 dead and 708 alive). The variables that presented a significant difference (p<0.05) between the living and dead group were age, the presence of arterial hypertension, and the total number of surgeries (*See* **Table 3**).

**Table 3:** Baseline bivariate analysis of mortality in patients with non-functioning pituitary adenomas.

Variable	Alive (n=708)	Dead (n=41)	P
Female, No. (%)	325 (45.90)	22 (53.65)	0.33
Age, median [IQR] years	60 [49-69.28]	68 [57-89]	<0.001
Age <60, No. (%)	363 (48.72)	15 (36.59)	0.13
Any family history, No. (%)	440 (62.14)	26 (63.41)	0.87
Lag-time at diagnosis, median [IQR] months	9.43 [2.63-22.35]	6.37 [2-13.2]	0.24
*Comorbidities*			
Systemic arterial hypertension, No. (%)	189 (26.69)	21 (51.22)	0.001
Dyslipidemia, No. (%)	198 (27.96)	7 (17.07)	0.12
Diabetes type 2, No (%)	104 (14.68)	9 (21.95)	0.20
*Clinical data*			
Campimetric deficit, No. (%)	627 (88.55)	34 (82.93)	0.27
Initial panhypopituitarism, No. (%)	141 (19.91)	9 (21.95)	0.75
Giant adenoma, No. (%)	134 (18.92)	4 (9.76)	0.14
Headache, No. (%)	104 (14.68)	9 (21.95)	0.20
Stroke, No. (%)	46 (6.49)	3 (7.32)	0.83
Ophthalmoplegia, No. (%)	27 (3.81)	1 (2.44)	0.65
Endocranial hypertension, No. (%)	22 (3.10)	1 (2.44)	0.80
Incidentaloma, No. (%)	56 (7.90)	6 (14.63)	0.12
Diabetes Insipidus, No. (%)	122 (17.23%)	6 (14.63%)	0.66
*Tumor characteristics*			
Dm cephalocaudal, median [IQR] cm	3 [2.5-3.5]	3 [2.5-3.5]	0.91
Dm transverse, median [IQR] cm	2.6 [2.1-3]	2.6 [2.13-3.1]	0.95
Dm anteroposterior, median [IQR] cm	2.5 [2.1-3]	2.5 [2.3-3]	0.55
Initial tumor volume, median [IQR] cm3	10.21 [6.46-14.14]	10.21 [6.7-16.38]	0.95
Invasion to neighboring structures, No. (%)	478 (67.51)	27 (65.85)	0.82
*Treatments*			
Surgical, No. (%)	487 (68.78)	38 (92.68)	0.001
Radiotherapy, No. (%)	147 (20.76)	7 (17.07)	0.57

### 3.4 Analysis of mortality predictors

In a multivariate Cox proportional hazards analysis, an adjusted Hazard Ratio (HR) of 2.19 (95% CI 1.13-4.26, p= 0.020) was observed for arterial hypertension and 1.32 (95% CI 1.00-1.75, p=0.045) for the total number of surgeries. The rest of the variables had no statistically significant association with mortality (**Table 4**). In the Kapplan Meyer survival analysis, a statistical difference was found with >60 years and arterial hypertension. (See **Supplementary Figures 1–4**).

**Table 4:** Cox proportional hazards analysis of mortality in patients with NFPA and adjusted analysis with factors associated with mortality (Age ≥60 years, sex, comorbidities, total number of surgeries, initial tumor volume, giant adenoma at diagnosis).

Variable	HR crude	95% CI	P	Adjusted HR	95% CI	P
Age ≥ 60 years	2.01	1.05 – 3.82	0.03	1.63	0.83 - 3.18	0.14
Sex	1.13	0.6 - 2.09	0.69	1.17	0.62 - 2.19	0.61
Arterial hypertension	2.65	1.43 – 4.89	0.002	2.19	1.13 - 4.26	0.02
Total number of surgeries	1.33	1.01- 1.75	0.04	1.32	1.00 - 1.75	0.04
Initial tumor volumen	0.99	0.97 - 1.01	0.69	0.99	0.99 - 1.00	0.81
Diabetes type 2	1.57	0.75 - 3.29	0.23	1.27	0.57 – 2.80	0.55
Giant adenoma	0.44	0.15 - 1.25	0.12	0.47	0.16- 1.35	0.16

## 4 Discussion

This retrospective study was aimed at answering two fundamental questions. The first one was related to the risk of increased mortality in patients with NFPA compared to the general population in Mexico, where we found evidence of it being 8.86 times higher. Secondly, we analyzed some predictors of higher mortality in our patients with NFPA, where systemic arterial hypertension and a greater number of surgeries increased the risk of death.

Regarding the standardized mortality rate associated with having NFPA, a Danish study conducted by Nielsen et al, who evaluated 192 patients with a follow-up of 13.1 years on average,100% treated with surgery and 22% with radiotherapy, found that the overall SMR for NFPA was 1.21 (95% CI 0.93-1.59) (14). In an analysis including 1014 UK patients, of which 57% included patients with NFPA, Tomlinson et al. found that the overall SMR in this population was 1.70 (99% CI 1.34-2.15, p=0.02) compared to the general population. The causes of excess mortality attributable in this cutoff were cardiovascular, respiratory and cerebrovascular causes (24). In a study of 546 patients, with a mean follow-up of 96 months, Ntali et al., found an increased mortality (SMR 3.6, 95% CI 2.9-4.5, p<.0.001) of patients with NFPA, especially for those who were operated on before 1990 (SMR 4.7, 95% CI 2.7-7.6, p<.0.001), with tumor regrowth (SMR 3.9, 95% CI 2.9-5.1, p<0.001), treated with adjuvant radiotherapy (SMR 4.1, 95% CI 2.7-6.0, p<0.001) and those who did not receive treatment of GH deficiency (SMR 3.9, 95% CI 2.8-5.2, p<0.001) (11).

Olsson et al. did an analysis including 2795 patients with NFPA, with a mean follow-up of 84 months. They found an overall SMR of 1.10 (95% CI 1.00-1.20, p=0.047) compared to the general population, an SMR of 2.68 (95% CI 1.23-5.09, p=0.015) for patients <40 years, in women with NFPA of 1.29 (95% CI 1.11-1.48, p=0.0008), in those diagnosed with any disease of the circulatory system of 1.21 (95% CI 1.06-1.38, p=0.007) and in those who received only radiotherapy treatment of 2.67 (95% CI 1.79-3.84, p<0.001) (13). In a later study, the same authors found that in the women’s group, mortality decreased throughout follow-up (SMR 1.50, 95% CI 1.21-1.84, p=0.0004 vs. SMR 1.16, 95% CI 0.83-1.58, p=0.38) (15).

In Mexico the mortality rate in general population is approximately 6.17 per 1000 inhabitants, where the main causes of death are represented by cardiovascular comorbidities, diabetes and malignant tumors (25). In the case of type 2 diabetes, the SMR for the general population is 2.28 (99% CI 2.214-2.353, p<0.0001) (20–23). In our cohort of patients with NFPA, we found that the overall SMR was of 8.86 (99% CI 5.92-13.25), SMR in <60 years was 13.77 (99% CI 7.23-26.23). In men it was 7.08 (99% CI 3.92-12.80), and in women it was 11.11 (99% CI 6.41-19.24). Regarding comorbidities, the SMR for arterial hypertension was 6.59 (99% CI 3.75-11.56) and in type 2 diabetes, it was 4.63 (99% CI 1.96-10.93) (*see* **Table 2**).

In a random-effects meta-analysis, Bioletto et al. included 11 studies of which 5 reported SMR (>20 000 patients). Mortality in patients with NFPA was found to be higher compared to the general population (SMR 1.57, 95% CI 1.20-1.99, p<0.001); in women (SMR 1.57, 95% CI 0.91-2.41, p=0.10) and in <40 years (SMR 3.19, 95% CI 2.50-3.97p<0.01) (26).

In our study, as in the studies of other authors, we found that the increase in mortality was more strongly associated with female gender, younger age, and the presence of cardiovascular comorbidity (arterial hypertension) and diabetes.

### 4.1 Predictors of mortality

Ntali et al. found that the significant predictors of mortality in univariate Cox proportional hazard analysis were age at diagnosis (HR 1.10, 95% CI 1.08-1.13, p<0.001), radiotherapy (HR 2.2, 95% CI 1.31-3.73, p<0.001) and tumor regrowth (HR 1.99, 95% CI 1.11-3.56, p=0.02), remaining statistically significant in multivariate analysis adjusted only for age at diagnosis (HR 1.10, 95% CI 1.07-1.13, p<0.001) (11). Olsson et al. found that in both genders, age at diagnosis (Men [HR 1.12, 95% CI 1.10-1.13, p<0.0001] Women [HR 1.13, 95% CI 1.11-1.15, p<0.0001]), age at diagnosis ≤40 years (Men [HR 3.47, 95% CI 1.28-9.42, p=0.015], Women [HR 4.20, 95% CI 1.32-13.32, p=0.015]) and treatment with radiotherapy (Men [HR 1.99, 95% CI 1.15-3.42, p=0.014] Women [HR 2.81, 95% CI 1.63-.4.83, p<0.0001]) behaved as potential predictors of mortality (13). However, in a subsequent study, the same group included patients diagnosed with NFPA between 1997-2011, where radiotherapy was found to have no effect on mortality (27). Van Varsseveld et al. who conducted a study involving 806 patients, with a median follow-up of 120 months, confirmed that radiotherapy had no effect on patient mortality (HR 0.89, 95% CI 0.46-1.58, p=0.62) (16). In our series, the mean median follow-up was 60.2 (min-max: 32.9-120.6), crude analysis of predictors of mortality showed that age ≥60 years had a HR of 2.01 (95% CI 1.05-3.82), presence of arterial hypertension a HR of 2.65 (95% CI 1.43-4.89) and total number of surgeries had a HR of 1.33 (95% CI 1.01-1.75), all being statistically significant; while in the adjusted multivariate analysis, the total number of surgeries (HR 1.32, 95% CI 1.00-1.75) and arterial hypertension (HR 2.19, 95% CI 1.13-4.26) remained statistically significant. Factors previously identified in other studies (12, 28) such as pituitary apoplexy and tumor regrowth, did not emerge as significant predictors in our cohort.

As in other survival series published in the literature by different authors in NFPA, the high mortality rate in our series was a probable consequence of the sum of different predictor variables, such as age at diagnosis (younger patients), female gender and arterial hypertension, the latter reaching a statistically significant difference. Indeed, the SMRs in our population are higher compared to those published in other centers, which could be due to different factors such as the number of surgeries performed on patients, secondary to a higher frequency of recurrence and/or persistence of pituitary tumor events. In a sub-analysis (data not shown) of 77 patients with NFPA, it was found that patients who died were carriers of silent somatotropic and lactotropic tumors (33% in deceased vs. 11.7% in living patients). Similarly, perhaps the fact that the patients who died had a greater number of surgeries implied a greater number of hospitalizations and prolonged stays, of intrahospital infections (respiratory, neuro-infection, etc.) and hemorrhagic complications during surgery, which increased mortality.

We believe that the results of this study could inform strategies for disseminating information about pituitary tumour disease to the economically active population. This would enable tumours to be detected earlier in symptomatic patients, reducing the time taken to make a diagnosis and the waiting time for timely surgical treatment, with fewer associated complications.

### 4.2 Limitations and strengths of the study

Strengths: Our study consists of a well-characterized, structured and captive cohort with adequate follow-up over time. This cohort is representative of the population in Mexico, as the study is conducted at a third-level care centre. The large number of patients contributes to better external validity.

Limitations: As this is a retrospective study, it may be difficult to access other data affecting mortality in patients with NFPAs, such as the number of patients with infections and pre-, intra- and postoperative complications. Additionally, selection bias may be present as the study was performed on captive patients in a tertiary care hospital, increasing the probability of type 2 error. The use of non-probability sampling increases the likelihood of a type 1 error.

## 5 Conclusions

This retrospective study of our NFPA patient cohort found evidence of higher mortality than published for other populations. This was associated with younger age, female gender, and the presence of comorbidities such as type 2 diabetes and arterial hypertension. Our study found a greater probability of death in the presence of predictors such as arterial hypertension and the total number of surgeries.

## Data Availability

The original contributions presented in the study are included in the article/**Supplementary Material**. Further inquiries can be directed to the corresponding author.
