# Supplementary material for: Increased mortality in patients with non-functioning pituitary tumors: a study in a tertiary center
Source: Front Endocrinol (Lausanne). 2025 Aug 4;16:1653376. doi: 10.3389/fendo.2025.1653376 (PMC12358282; doi:10.3389/fendo.2025.1653376)
Supplement: Supplementary file 1 [file DataSheet1.docx]

*Supplementary Table 1. Details of hormone assays: Measurement intervals, precision and mean concentrations.*

| **Hormone** | **Measuring range** | **Intra-assay precision (%)** | **Interassay precision (%)** | **Average concentration** |
| --- | --- | --- | --- | --- |
| **Cortisol** | 0.054-63.4 µg/dL (up to 634 µg/dL diluted 1:10) | 1.10% | 1.80% | 9.90 µg/dL |
| **TSH** | 0.005-100 µIUI/mL (up to 1000 µIUI/mL diluted 1:10) | 0.70% | 2.20% | 4.09 µUI/mL |
| **FT4** | 0.5-100 pmol/L | 1.10% | 2.40% | 22.6 pmol/L |
| **Testosterone** | 0.025-15 ng/mL | 1.60% | 3.20% | 2.09 ng/mL |
| **Estradiol** | 5-3000pg/mL | 1.4% | 1.8% | 191 pg/mL |
| **LH** | 0.3-200 mIU/mL | 1.00% | 1.10% | 63.4 mIU/mL |
| **FSH** | 0.3-200 mIU/mL | 2.10% | 3.80% | 75.3 mIU/mL |
| **Prolactin** | 0.094-470 ng/mL | 1.80% | 2.70% | 28.1 ng/mL |
