# Supplementary material for: Increased mortality in patients with non-functioning pituitary tumors: a study in a tertiary center
Source: Front Endocrinol (Lausanne). 2025 Aug 4;16:1653376. doi: 10.3389/fendo.2025.1653376 (PMC12358282; doi:10.3389/fendo.2025.1653376)

**Supplementary Figure.**

*Suplemmentery Figure 1. Survival analysis in a cohort of nonfunctioning pituitary adenoma by age.*

*Alt text: Chart comparing survival over time across different age groups, showing lower survival in individuals over 60 years.*


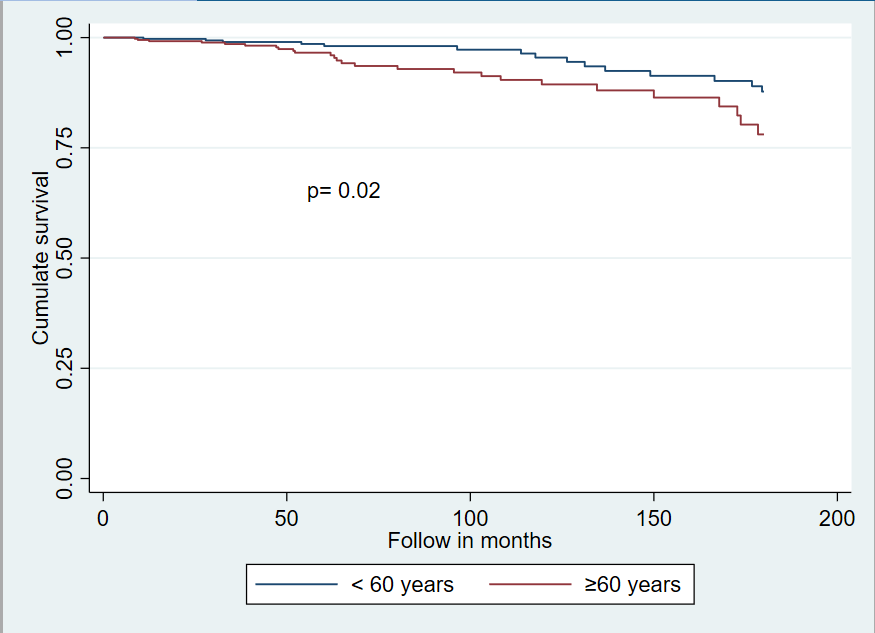


*Supplementary Figure 2. Survival analysis in a cohort of non-functioning pituitary adenoma by gender.*

*Alt text: Chart comparing survival over time across gender, women show higher survival early on, but slightly lower survival by the end of follow up.*


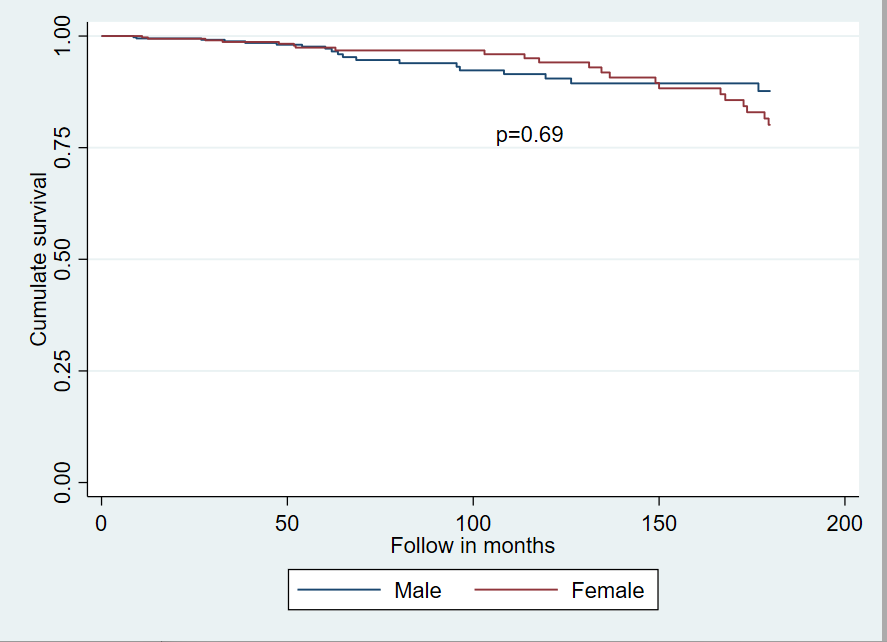


*Supplementary Figure 3. Survival Analysis in a cohort of nonfunctioning pituitary adenoma by blood pressure.*

*Alt text: Chart comparing survival over time across presence of arterial hypertension, showing lower survival in individuals with arterial hypertension.*


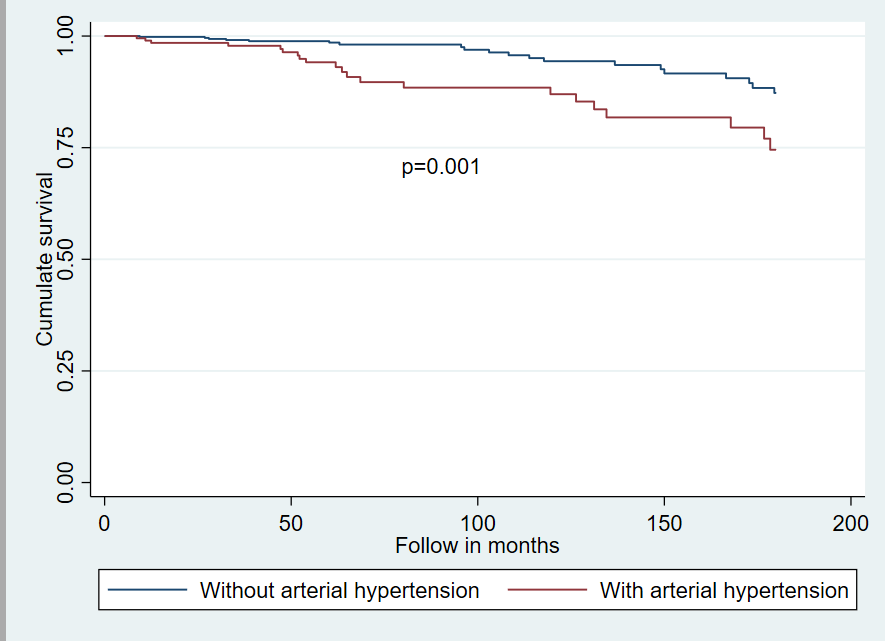


*Supplementary Figure 4. Survival Analysis in a cohort of nonfunctioning pituitary adenoma by number of surgeries.*

*Alt text: Chart comparing survival over time across the number of surgeries, showing lower survival in individuals who underwent a higher number of surgeries.*


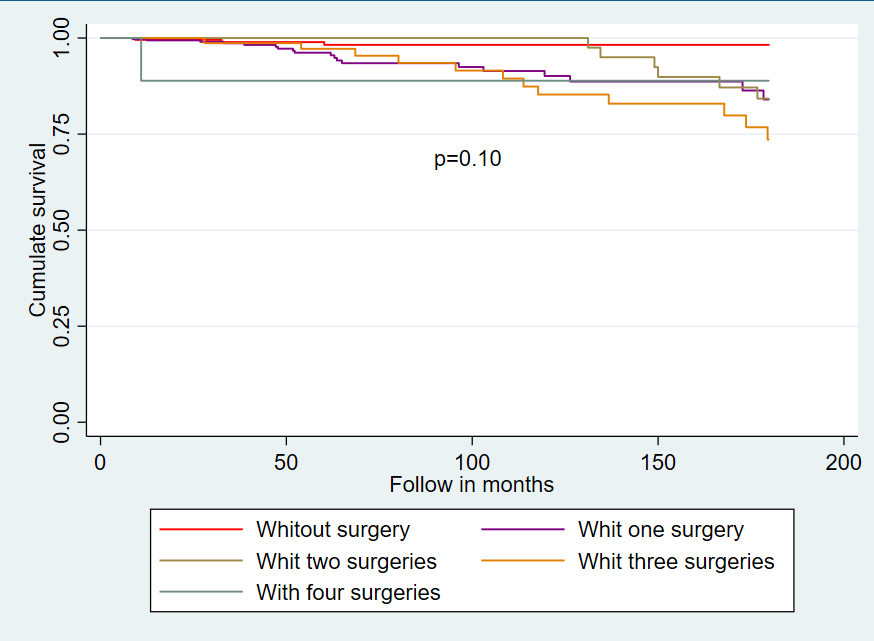

Supplement: Supplementary file 2 [file DataSheet2.docx]
